# Unmasked Behavioral Disturbance Following Traumatic Brain Injury in an Adult With Previously Undiagnosed Sotos Syndrome

**DOI:** 10.7759/cureus.113036

**Published:** 2026-07-20

**Authors:** Koji Obara

**Affiliations:** 1 Neurology, National Hospital Organization Akita National Hospital, Yurihonjo, JPN

**Keywords:** behavioral disturbance, neurodevelopmental disorder, propranolol, sotos syndrome, traumatic brain injury(tbi), valproate

## Abstract

Sotos syndrome is a rare autosomal dominant disorder characterized by childhood overgrowth, including tall stature and macrocephaly, distinctive facial appearance, and neuropsychiatric manifestations. These features include intellectual disability and a broad spectrum of behavioral disturbances. Traumatic brain injury (TBI) is also known to cause various neuropsychiatric symptoms, including behavioral disturbances, even in individuals without underlying medical conditions. However, the influence of pre-existing neurodevelopmental disorders such as Sotos syndrome on behavioral outcomes following TBI remains incompletely understood.

We report the case of a 35-year-old man with previously undiagnosed Sotos syndrome who developed severe behavioral disturbances following TBI. Based on his history of childhood overgrowth and characteristic dysmorphic facial features, Sotos syndrome was suspected and subsequently confirmed by genetic testing, which demonstrated a heterozygous whole-gene deletion of the causative gene, *NSD1*. Initial treatment with yokukansan and olanzapine did not improve his symptoms. However, subsequent treatment with valproate and propranolol resulted in marked improvement. The total Neuropsychiatric Inventory Questionnaire (NPI-Q) score decreased from 43 to 15 after one year of treatment.

This report suggests that TBI may unmask or exacerbate behavioral disturbances in individuals with underlying neurodevelopmental disorders such as Sotos syndrome. In addition, a combination of valproate and propranolol may represent a potential therapeutic option for managing severe behavioral symptoms following TBI, although further accumulation of cases is required to establish its efficacy.

## Introduction

Sotos syndrome is a rare autosomal dominant disorder characterized by childhood overgrowth (including tall stature and macrocephaly), distinctive facial appearance, and neuropsychiatric manifestations [1]. These neuropsychiatric features include intellectual disability and a broad spectrum of behavioral disturbances, such as autistic traits, stereotyped behaviors, repetitive language, impulsivity, hyperactivity, irritability, aggression, and anxiety [1-3].

Traumatic brain injury (TBI) is also known to cause various neuropsychiatric symptoms, including behavioral disturbances [4]. Even individuals without underlying medical conditions may develop severe behavioral disturbances following TBI [4,5]. However, the influence of pre-existing neurodevelopmental disorders on behavioral outcomes following TBI remains incompletely understood. Management of neuropsychiatric symptoms following TBI is often challenging [6,7]. Although several pharmacological agents have been used, evidence supporting their efficacy remains limited [6,7]. Previous studies have suggested that valproate and propranolol may be effective in reducing agitation and aggression following TBI. However, the overall level of evidence is weak [6,7]. In addition, little is known about the impact of pre-existing neurodevelopmental disorders on behavioral outcomes following TBI.

We report what is, to our knowledge, the first genetically confirmed adult case of previously undiagnosed Sotos syndrome presenting with severe behavioral disturbances following TBI. This report highlights the potential interaction between TBI and underlying neurodevelopmental vulnerability. Behavioral symptoms subsequently improved with treatment with valproate and propranolol.

## Case presentation

A 35-year-old man visited our institution with his mother because of severe behavioral disturbances. He had been born at term via cesarean section due to cephalopelvic disproportion and fetal distress to healthy, non-consanguineous parents. His birth weight was 3,350 g, length 52 cm, and head circumference 35.0 cm. He was noted to have a cleft palate, which was surgically repaired at three months of age. His developmental milestones were mildly delayed; he achieved head control at four months and independent ambulation at 18 months. From approximately two years of age, his height and weight consistently exceeded the mean by more than +2 standard deviations. Due to intellectual disability, he was issued a Japanese Rehabilitation Certificate (category B1; IQ 36-50) and attended a special support school. Before the injury described below, he was able to communicate with family members and enjoyed social interaction. Although he occasionally exhibited temper outbursts, he was not hyperactive and was able to travel with his family without difficulty.

At the age of 33 years, he fell from a second-floor window at home, resulting in an acute subdural hematoma and a pelvic fracture. According to the referral records from the previous hospital, his level of consciousness on admission was a Japan Coma Scale score of II-10 and a Glasgow Coma Scale score of 11 (E3V3M5). CT performed the following day revealed bilateral frontal lobe contusions. Because the subdural hematoma remained stable, he was managed conservatively without neurosurgical intervention and received levetiracetam 2,000 mg/day for seizure prophylaxis. He was hospitalized for approximately two months. Following the injury, he developed marked behavioral abnormalities, including inappropriate licking of calendar numbers and flushing batteries down the toilet. He also exhibited disinhibition and impulsivity, such as performing somersaults indiscriminately, sudden physical aggression toward others, and hyperphagia. His body weight increased by approximately 20 kg after the injury.

On his first visit to our institution at age 35, his height was 170 cm, body weight was 78 kg, and head circumference was 59 cm. He exhibited characteristic dysmorphic facial features, including a broad forehead, long face, pointed chin, and mildly down-slanted palpebral fissures. On examination, he responded to simple questions in a loud, rapid voice. However, echolalia and perseverative responses to prior questions were frequently observed. He was restless and easily distracted during the examination. He occasionally displayed sudden, impulsive behaviors, such as forcefully striking the desk with both hands or grabbing the examiner’s hand or name tag. He was unable to imitate simple hand gestures.

**Figure 1:**
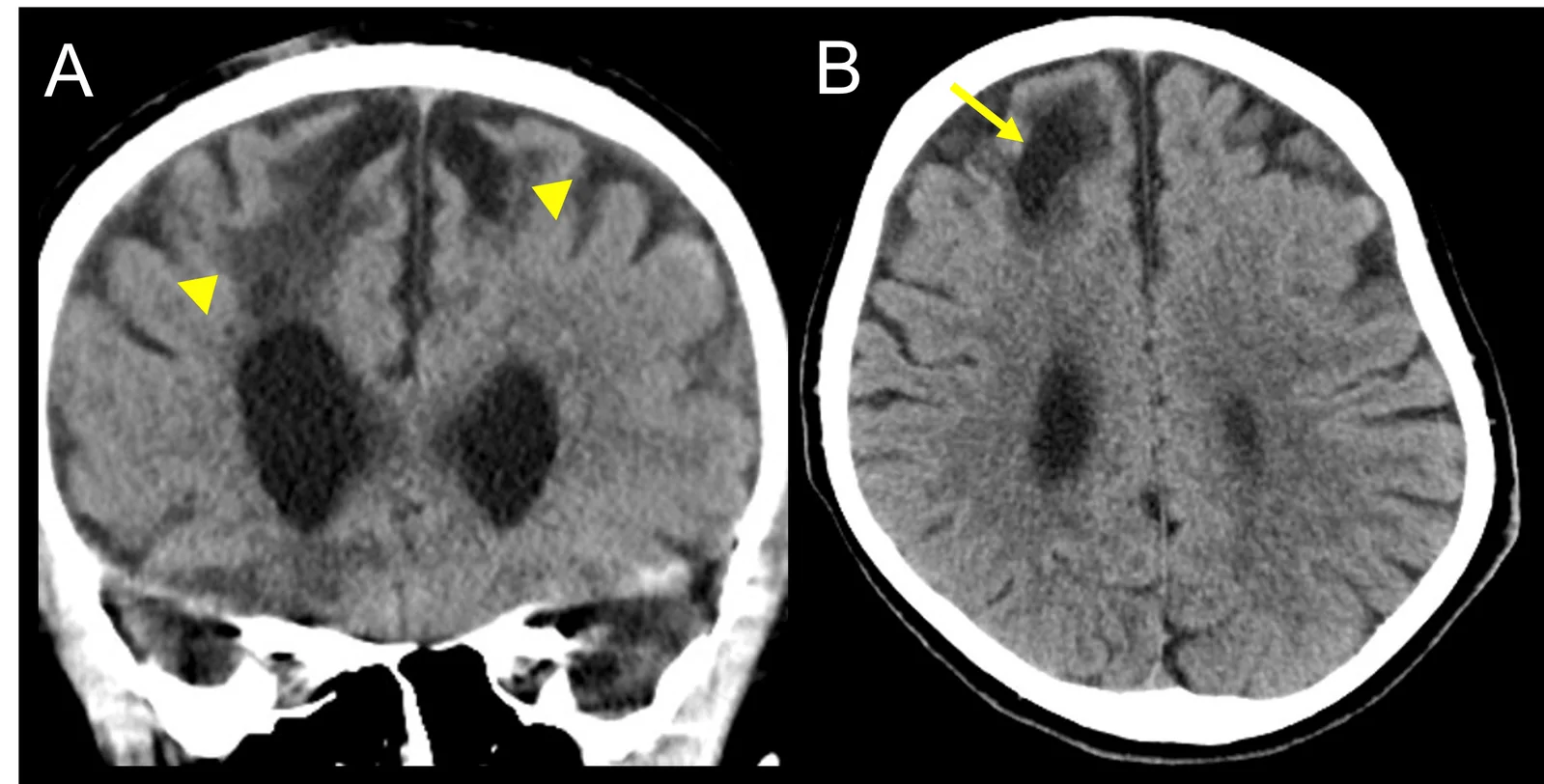
Head CT obtained two years after traumatic brain injury (A) Coronal reconstructed image. (B) Axial image. Low-density lesions involving the cortex and subcortical white matter of the bilateral frontal lobes, predominantly affecting the superior frontal gyri (arrowheads). Cystic changes are observed within the superior frontal lesions, with greater involvement on the right side (arrow) CT: computed tomography

Neurological examination revealed a dragging gait of the right lower extremity. Deep tendon reflexes were increased in the lower right limb, and a Babinski sign was present on the right side. Laboratory investigations, including complete blood count, liver and renal function tests, thyroid function, uric acid, plasma ammonia, and vitamin B12 levels, were all within normal limits. G-banded karyotyping at approximately 550-band resolution showed a male karyotype (46, XY) with no detectable structural chromosomal abnormalities. A head CT scan performed two years after the injury revealed low-density lesions involving the cortex and subcortical white matter of the bilateral frontal lobes, predominantly affecting the superior frontal gyri. The bilateral superior frontal lesions were accompanied by cystic changes, particularly on the right side (Figure 1).

**Figure 2:**
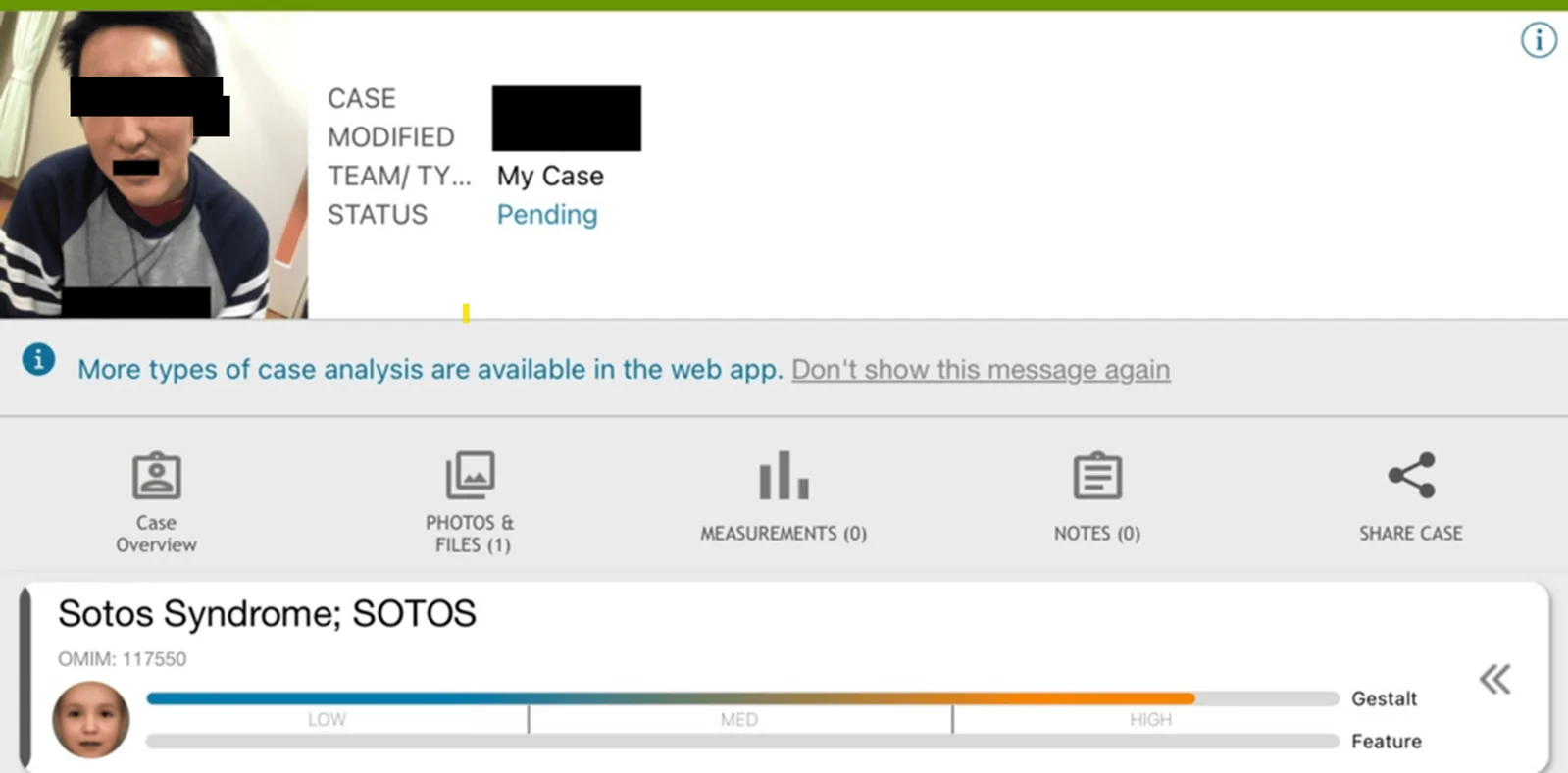
The analysis of the patient's face using Face2Gene The patient's face had a broad forehead, long face, pointed chin, and mildly downslanting palpebral fissures. Face2Gene showed that the patient's face had a high gestalt score for Sotos syndrome

To investigate the etiology of the patient’s facial dysmorphism and intellectual disability, further analyses were performed. First, facial analysis using Face2Gene (FDNA Inc., Boston, MA) demonstrated a high gestalt score suggestive of Sotos syndrome (Figure 2) [8].

**Figure 3:**
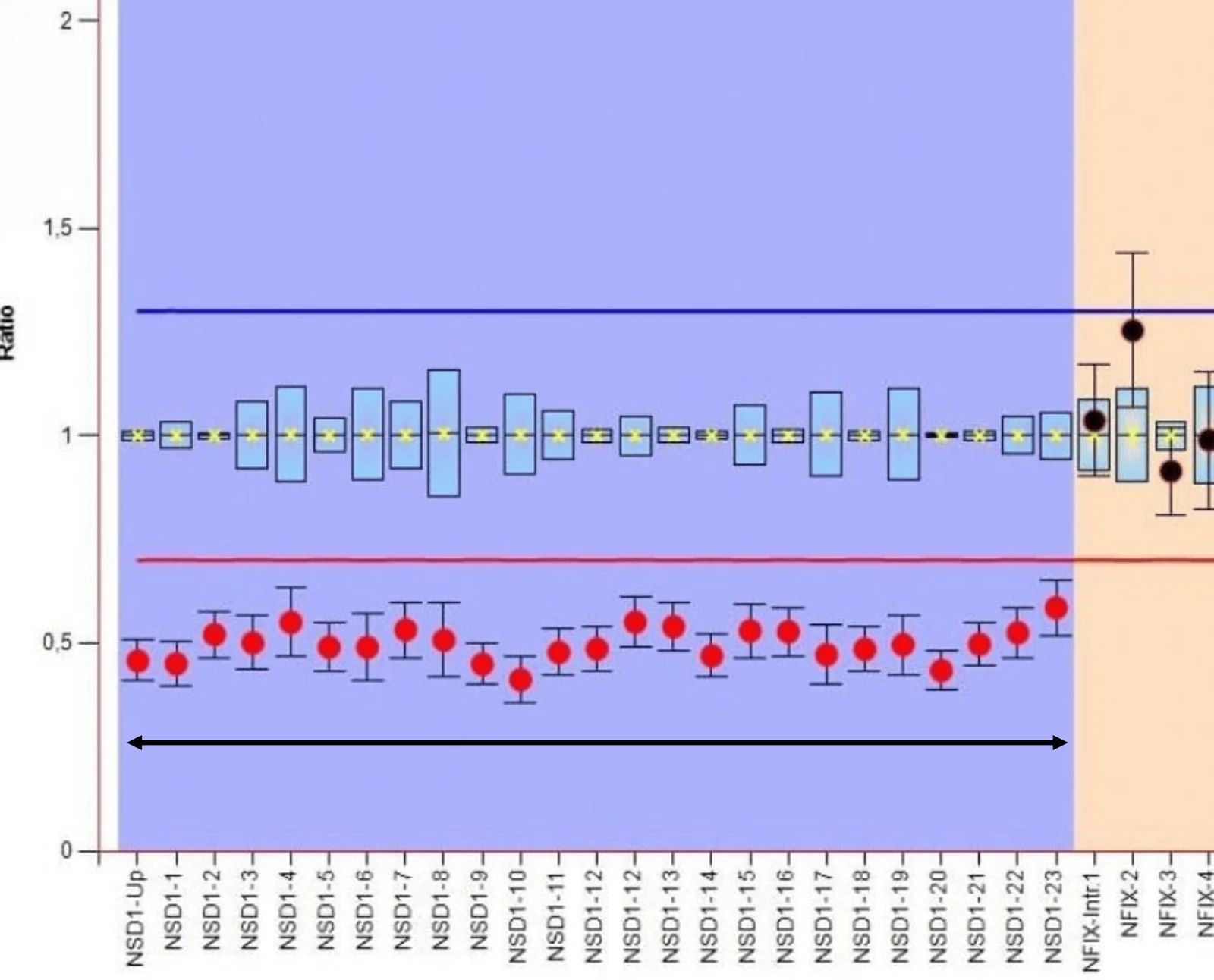
Multiplex ligation-dependent probe amplification (MLPA) of the patient The result shows a heterozygous whole-gene deletion of *NSD1*

Subsequently, genomic DNA was extracted from peripheral blood leukocytes using standard methods. All coding exons and exon-intron boundaries of the *NSD1* (nuclear receptor-binding SET domain protein 1) gene were analyzed using next-generation sequencing (NextSeq 2000; Illumina Inc., San Diego, CA). This analysis suggested a heterozygous large deletion involving the *NSD1* gene. Therefore, multiplex ligation-dependent probe amplification (MLPA) was subsequently performed and confirmed a heterozygous whole-gene deletion of *NSD1*, establishing the diagnosis of Sotos syndrome (Figure 3).

Following his initial visit to our institution, he was treated with yokukansan at a dose of 7.5 g/day; however, his family reported worsening irritability. Subsequently, treatment with olanzapine at 5 mg/day was initiated, but no significant improvement was observed. One year later, at the age of 36, levetiracetam was discontinued and replaced with valproate at 600 mg/day as an antiepileptic drug. Following this change, his irritability and abnormal behavior gradually improved. Furthermore, propranolol was initiated at 15 mg/day and titrated to 30 mg/day, leading to further amelioration of his behavioral symptoms. No structured behavioral therapy, cognitive rehabilitation, or other formal non-pharmacological intervention was implemented during the observation period. At present, he lives at home on weekdays and stays in a group home on weekends without major difficulties. He is independent in the basic activities of daily living.

The Neuropsychiatric Inventory Questionnaire (NPI-Q) (© JL Cummings, 1994, all rights reserved; permission for commercial use required; npiTEST.net) was administered at the initial visit before treatment initiation and repeated one year after initiation of valproate and propranolol, with the patient's mother serving as the respondent on both occasions [9]. As a result, the total NPI-Q score decreased from 43 to 15 (Table 1), indicating a marked improvement in neuropsychiatric symptoms.

**Table 1:** Changes in Neuropsychiatric Inventory Questionnaire (NPI-Q) scores before and after treatment with valproate and propranolol

NPI-Q domain	Severity before	Severity after	Distress before	Distress after
Delusions	1	1	0	0
Hallucinations	0	0	0	0
Agitation/aggression	3	1	4	1
Depression/dysphoria	0	1	0	0
Anxiety	3	1	4	2
Elation/euphoria	0	1	0	0
Apathy/indifference	0	0	0	0
Disinhibition	2	0	2	0
Irritability/lability	3	1	4	2
Motor disturbance	3	1	5	2
Nighttime behavioral disturbances	2	1	3	0
Appetite/eating disturbances	2	0	2	0
Total NPI-Q score	43	15	—	—

Written informed consent was obtained from the parent of the patient for the use of blood samples in the genetic analysis, a photograph of the patient’s face that was uploaded to the Face2Gene application for analysis, and publication of details of the medical case and any accompanying images, including photographs of the patient. The study was conducted in accordance with the principles of the Declaration of Helsinki.

## Discussion

This case highlights two important clinical points. First, severe behavioral disturbances developed following TBI in an adult with previously undiagnosed Sotos syndrome. Although a causal relationship cannot be established from a single case, the clinical course raises the possibility that TBI may have unmasked an underlying neurodevelopmental vulnerability. Sotos syndrome is a rare autosomal dominant overgrowth disorder caused primarily by haploinsufficiency of the *NSD1* gene located on chromosome 5q35 [1,10]. Neuropsychiatric manifestations are common and tend to be more severe in patients with 5q35 microdeletions than in those with intragenic *NSD1* variants [1-3,10]. In our patient, despite harboring a whole-gene deletion of *NSD1*, the relatively mild premorbid behavioral phenotype raises the possibility that TBI may have exacerbated an underlying neurodevelopmental vulnerability.

TBI is well known to cause significant changes in behavior, personality, and mood, particularly when the frontal lobes are affected [4]. Damage to these regions is frequently associated with disinhibition, impulsivity, and aggression, all of which were observed in this patient [4]. While TBI can induce neuropsychiatric symptoms even in individuals without prior conditions, the influence of pre-existing neurodevelopmental disorders on post-TBI behavioral outcomes remains incompletely understood [4,5]. To our knowledge, reports describing TBI in patients with Sotos syndrome or similar developmental disorders are scarce. Therefore, it remains unclear to what extent the underlying neurodevelopmental condition contributed to the severity of behavioral disturbances in this case.

The second important clinical point is that the patient’s post-TBI behavioral disturbances improved following treatment with valproate and propranolol. Management of neuropsychiatric symptoms after TBI is often challenging, and although various pharmacological agents-including antidepressants, antiepileptic drugs, and antipsychotics-have been used, robust evidence supporting their efficacy remains limited [4,6,7]. In clinical practice, treatment is typically individualized [6,7].

In this case, initial treatment with yokukansan and olanzapine did not improve symptoms, and irritability worsened with yokukansan. Subsequently, levetiracetam was discontinued and replaced with valproate, resulting in a gradual improvement in behavioral symptoms. Further improvement was observed after the addition of propranolol. Notably, improvements were particularly evident in agitation/aggression, disinhibition, irritability, and motor disturbance on the NPI-Q assessment. Previous studies have suggested that valproate may reduce agitation and aggressive behavior in TBI patients, while propranolol has been reported to decrease the intensity of agitation and reduce the need for physical restraints [6,7]. However, the available evidence for both agents remains limited due to small sample sizes, methodological heterogeneity, and a lack of controlled trials [6,7].

It is also important to consider that the observed improvement in this case may have been influenced by multiple factors, including discontinuation of levetiracetam - known to be associated with irritability and aggression [11] - as well as the natural course of recovery following TBI [12]. Therefore, the observed clinical improvement was likely multifactorial and cannot be attributed solely to valproate and propranolol. The therapeutic effects of these medications should therefore be interpreted with caution. In addition, although the NPI-Q provided a practical measure of behavioral symptoms in the present case, it has not been specifically validated for patients with TBI or Sotos syndrome. Because the patient's mother completed the NPI-Q, informant bias cannot be excluded and should also be considered when interpreting the observed clinical improvement.

## Conclusions

This report suggests that TBI may unmask or exacerbate behavioral disturbances in individuals with underlying neurodevelopmental disorders such as Sotos syndrome. In addition, a combination of valproate and propranolol may be a potential therapeutic option for managing severe behavioral symptoms following TBI, although further accumulation of cases is required to establish its efficacy. As a single-case observation, this report is hypothesis-generating, and causal relationships among TBI, underlying Sotos syndrome, and the observed therapeutic response cannot be established.

## References

[1] Ocansey S, Cole TRP, Rahman N, Tatton-Brown K (2025). Sotos Syndrome. https://www.ncbi.nlm.nih.gov/books/NBK1479/.

[2] Lesinskiene S, Montvilaite R, Pociute K, Matuleviciene A, Utkus A (2024). Neuropsychiatric aspects of Sotos syndrome: explorative review building multidisciplinary bridges in clinical practice. J Clin Med.

[3] Lane C, Milne E, Freeth M (2016). Cognition and behaviour in Sotos syndrome: a systematic review. PLoS One.

[4] Torregrossa W, Raciti L, Rifici C (2023). Behavioral and psychiatric symptoms in patients with severe traumatic brain injury: a comprehensive overview. Biomedicines.

[5] Tate RL (2003). Impact of pre-injury factors on outcome after severe traumatic brain injury: does post-traumatic personality change represent an exacerbation of premorbid traits?. Neuropsychol Rehabil.

[6] Robert S (2020). Traumatic brain injury and mood disorders. Ment Health Clin.

[7] Williamson D, Frenette AJ, Burry LD (2019). Pharmacological interventions for agitated behaviours in patients with traumatic brain injury: a systematic review. BMJ Open.

[8] Pantel JT, Hajjir N, Danyel M (2020). Efficiency of computer-aided facial phenotyping (DeepGestalt) in individuals with and without a genetic syndrome: diagnostic accuracy study. J Med Internet Res.

[9] Cummings JL, Mega M, Gray K, Rosenberg-Thompson S, Carusi DA, Gornbein J (1994). The neuropsychiatric inventory: comprehensive assessment of psychopathology in dementia. Neurology.

[10] Tatton-Brown K, Douglas J, Coleman K (2005). Genotype-phenotype associations in Sotos syndrome: an analysis of 266 individuals with NSD1 aberrations. Am J Hum Genet.

[11] Ramírez-Guerrero S, Gaviria-Carrillo M, Suarez-Rubiano J (2026). Neuropsychiatric adverse effects associated with the use of levetiracetam in adults: a systematic review and meta-analysis. Med Princ Pract.

[12] Hart T, Benn EK, Bagiella E (2014). Early trajectory of psychiatric symptoms after traumatic brain injury: relationship to patient and injury characteristics. J Neurotrauma.

